# Enhancing oral health outcomes through public health policy reform

**DOI:** 10.3389/froh.2025.1604465

**Published:** 2025-06-09

**Authors:** Chukwuemeka L. Anyikwa, Chukwuebuka E. Ogwo

**Affiliations:** ^1^ College of Medicine, University of Nigeria, Ituku-Ozalla Enugu, Nigeria; ^2^ Harvard School of Dental Medicine, Boston, MA, United States

**Keywords:** oral health policy, sustainability, health equity, disease burden, preventive strategies

## Abstract

This article explores the transformative potential of public health policies to improve oral health outcomes through the integrated application of three fundamental pillars: sustainability, equity in healthcare access, and the reduction of oral health disease burden. By examining the interplay of these pillars, the discussion proposes strategies that not only enhance preventive measures and accessibility to dental care but also foster long-term, sustainable improvements in population oral health. The framework presented herein is intended to guide policymakers in creating evidence-based interventions that address disparities and mitigate the growing burden of oral diseases globally.

## Introduction

Oral health remains a critical component of overall well-being, yet persistent disparities and a significant disease burden continues to affect diverse populations (1). The need for robust public health policies is paramount, particularly as oral diseases contribute substantially to global morbidity and socioeconomic inequities (2). This article leverages the pillars of sustainability, equity healthcare access, and oral health disease burden to frame public health policy interventions that promote prevention, improve access to care, and achieve equitable health outcomes.

To begin with, sustainability in oral health policy is essential for ensuring that interventions are not only effective in the short term but also resilient in the face of evolving public health challenges. Sustainable strategies involve the development of infrastructure that supports continuous improvement in service delivery, the implementation of preventive programs that reduce the incidence of oral diseases, and the adoption of practices that are both economically and environmentally viable (3). Prioritizing sustainability enables policymakers to build resilient health systems that can withstand future challenges while adapting to dynamic demographics and addressing the diverse needs of communities (4).

Equally important is the pillar of equitable access to oral healthcare. Despite advancements in dental care, significant gaps remain in access to quality oral health services (5). These disparities are often rooted in socioeconomic factors, geographic barriers and sometimes, cultural differences that hinder certain groups from receiving adequate care (6, 7). Policies aimed at promoting equity in care must address these issues head-on by expanding access to dental services through community outreach programs, mobile clinics, and teledentistry (8). Furthermore, investing in workforce development initiatives that ensure an adequate supply of trained dental professionals in underserved areas and removing barriers to access while ensuring that every individual receives timely and effective care allow public health policies to play a pivotal role in reducing oral health inequities (9–11).

The burden of oral diseases, including dental caries, periodontal diseases, and oral cancers, continues to escalate globally, placing an immense strain on healthcare systems (1, 2). The direct and indirect costs associated with treating advanced stages of these diseases further exacerbate the socioeconomic divide (12). Effective public health policies must therefore prioritize early intervention and prevention (13). This includes implementing evidence-based community programs, such as water fluoridation, school-based oral health education and routine screenings, which are proven to reduce the prevalence of oral diseases (9, 14). Moreover, a data-driven approach to resource allocation can help identify high-risk populations and regions, ensuring that interventions are targeted where they are most needed and that the overall burden of disease is systematically reduced (15, 16).

## Sustainability: building resilient oral health systems

Sustainability in oral health also means building partnerships across sectors and disciplines to ensure a well-coordinated approach to public health challenges (17). Collaborations between government agencies, private dental care providers, academic institutions, and community organizations are essential for pooling resources, sharing best practices and fostering innovations that can lead to more resilient oral health systems (9, 18). Such partnerships facilitate the development of integrated care models that not only respond to current needs but also anticipate future challenges by incorporating emerging technologies and evolving healthcare delivery methods (19–21).

Sustainable oral health policies should embed mechanisms for regular assessment and feedback, allowing for iterative improvements in strategy and execution (22). Setting measurable goals and performance indicators enables policymakers to track progress, identify areas for improvement and ensure that investments generate long-term benefits (23). This continuous cycle of evaluation and adjustment helps maintain a dynamic system that remains responsive to changes in population demographics, disease patterns, and technological advancements (24).

Another critical aspect is the integration of environmental stewardship into oral health initiatives. As the healthcare sector increasingly prioritizes reducing its ecological footprint, oral health policies must incorporate eco-friendly practices, from sustainable procurement of dental materials to energy-efficient clinic operations, ensuring that healthcare delivery aligns with environmental goals while remaining economically viable and socially responsible (25, 26).

Specific national policies explicitly supporting green dentistry are limited. However, organizations like the European Federation of Periodontology and the FDI World Dental Federation offer guidelines and recommendations for sustainable dental practices within the dental field. These include energy efficiency measures (e.g., LED lighting, renewable energy), water conservation (e.g., low-flow faucets), and waste management (e.g., recycling, biodegradable materials). An overview of these sustainability strategies and their operational implications is provided in Table 1.

**Table 1:** Sustainable dental practice matrix: strategies, steps, and outcomes

Strategy	Practical steps	Expected benefit
**Sustainable procurement**	• Draft a “green” supplier list • Specify reusable over single-use where clinically safe	↓ Supply-chain emissions; ↑ supports eco-responsible vendors
**Waste segregation & recycling**	• Colour-coded bins for recyclables • Transition to digital x-rays	↓ Landfill waste; ↑ proper hazardous-waste handling
**Energy-efficient infrastructure**	• LED retrofit; motion sensors • Programmable HVAC controls	↓ Electricity costs; ↓ GHG emissions
**Water conservation**	• Low-flow fixtures; dry-vacuum systems • Regular leak detection	↓ Water usage; preserves local water resources
**Green sterilization & disinfection**	• Use sterilizers with water recapture • Choose low-toxicity disinfectants	↓ Chemical effluent; ↓ water demand
**Preventive & minimally invasive care**	• Risk-based recalls • Atraumatic Restorative Techniques (ART)	↓ Resource-intensive treatments; ↑ patient well-being
**Tele-dentistry integration**	• Remote triage and follow-ups via secure platforms • Patient self-photography for screening	↓ Travel-related footprint; ↑ access
**Environmental training & culture**	• Sustainability in CPD • Appoint “green champions”	↑ Staff engagement; continuous eco-auditing
**Environmental impact assessment**	• Annual audit of utilities and waste • Set and monitor SMART reduction targets	Data-driven progress with accountability
**Policy & professional advocacy**	• Propose sustainability metrics in licensing • Engage associations to reward environmentally friendly clinics	Embeds sustainability at system level and drives broad change

Sustainability in oral health is not a static goal but an ongoing process that requires vision, commitment, and collaboration, and by embedding these principles into public health policies, communities can ensure accessible quality dental care, minimize environmental impacts and maintain a system agile enough to meet society's evolving needs (9, 25, 27).

## Equity in access to oral healthcare: addressing disparities in oral health

**Figure 1:**
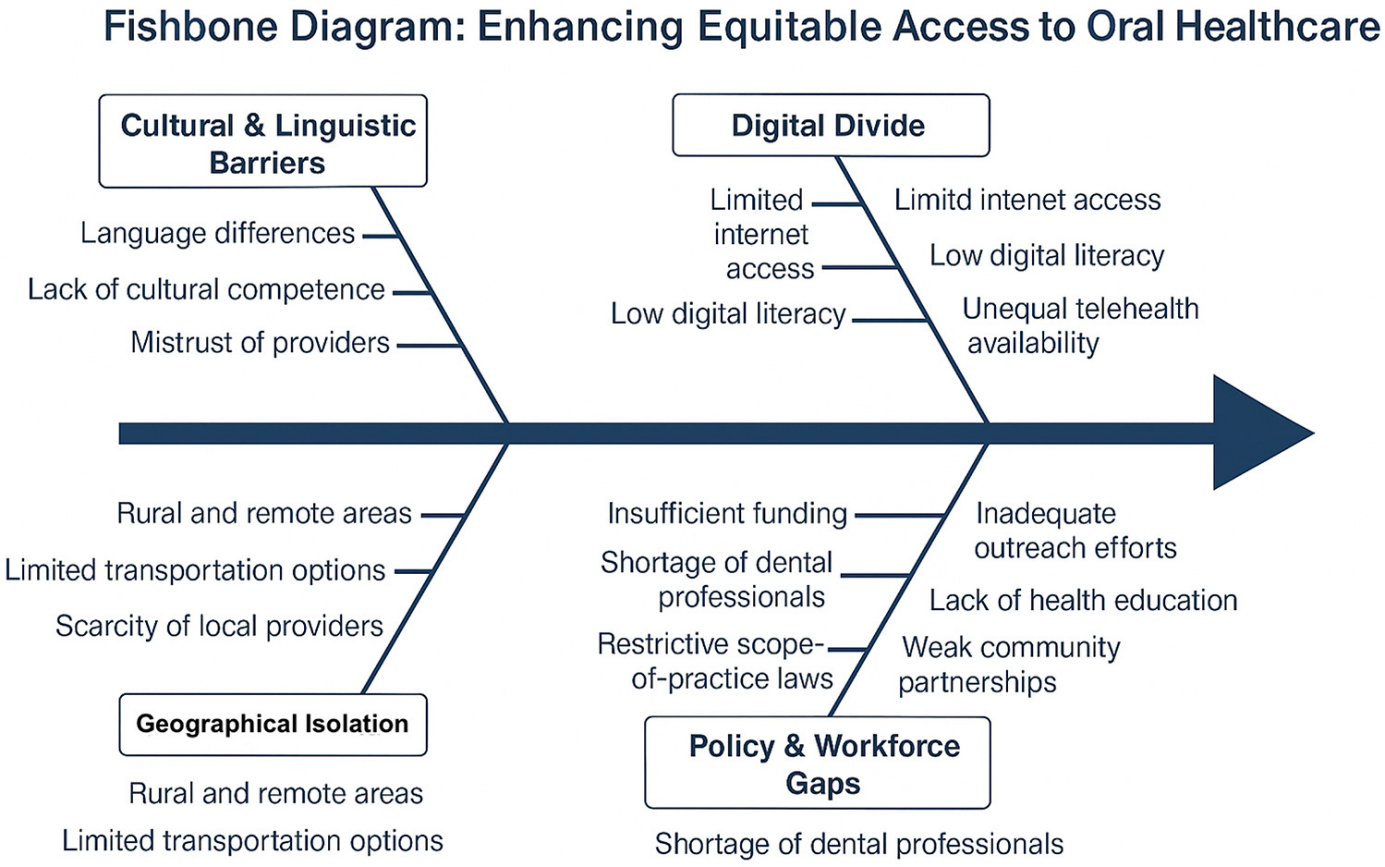
Fishbone model: enhancing equitable access to oral healthcare.

Equitable access to oral health care involves tailoring healthcare delivery to meet the unique cultural, linguistic and socioeconomic needs of diverse communities (28, 29). This multifactorial challenge is depicted in Figure 1, which outlines a Fishbone Model for Enhancing Equitable Access to Oral Healthcare. This means developing culturally competent care models that acknowledge and address specific barriers faced by minority and marginalized populations (30, 31). Such models involve training healthcare professionals in cultural sensitivity, integrating community health workers who share similar backgrounds with the target populations, and designing outreach programs that resonate with local values and practices (30, 32).

In addition, targeted policies must focus on reducing systemic barriers that limit access to quality dental care. This includes addressing financial obstacles through subsidized services or insurance schemes and overcoming logistical challenges by investing in transportation and digital connectivity for tele-dentistry initiatives (9). These strategies can bridge the gap between urban centers and rural or remote areas, ensuring that underserved populations receive the same standard of care as their more urban counterparts (33).

Moreover, fostering strong partnerships with community organizations, faith-based groups, and local leaders is crucial in disseminating health information and building trust (34). Community engagement not only increases awareness about the importance of preventive dental care but also encourages collective action toward creating supportive environments for oral health (9, 35).

## Oral health disease burden: prioritizing high-impact interventions that is population specific

The global burden of oral diseases, such as dental caries, periodontal diseases, and oral cancers, places immense pressure on healthcare systems worldwide. Among children and adolescents, dental caries remains the most prevalent condition, calling for preventive strategies such as school-based fluoride varnish programs, water fluoridation, and sugar intake regulation. In working-age adults, periodontal diseases dominate, requiring policies that promote oral hygiene education, smoking cessation initiatives, and early periodontal screening within workplaces and community health centers. For older adults, particularly in populations with high rates of tobacco and alcohol consumption, oral cancers present a significant threat, emphasizing the need for systematic oral cancer screenings embedded in primary care services (1, 2). Targeted policies for specific burdens and populations ensure that interventions are aligned with the actual patterns of disease, allowing for efficient resource allocation and greater public health impact. Effective public health policies must focus on targeted interventions backed by robust epidemiological research and demonstrated success (36). To identify and prioritize these burdens accurately, epidemiological data must be systematically gathered through comprehensive needs assessments which captures disease prevalence and service gaps through continuous surveillance systems that monitor oral health trends over time. Following this, implementing population-based preventive measures becomes imperative (37). In addition to prevention, early detection plays a critical role in mitigating the severity of oral diseases (1, 9). Integrating routine oral health screenings into general health check-ups allows for the early identification of potential issues, enabling prompt intervention before conditions escalate into more severe or life-threatening stages (38, 39). This proactive approach not only improves individual outcomes but also reduces the long-term economic burden on healthcare systems by preventing costly treatments and hospitalizations associated with advanced oral diseases (40).

Furthermore, the strategic allocation of resources based on robust epidemiological data is essential for targeting interventions effectively (41). This targeted approach ensures that preventive programs and treatment facilities are established in high-risk areas, thus maximizing the impact of interventions and promoting a more equitable distribution of healthcare services (4, 6, 40).

High-impact interventions should also incorporate innovative solutions such as community-based screening programs, mobile dental clinics, and tele-dentistry services (42). These innovations extend the reach of traditional healthcare systems, particularly in underserved rural and remote communities where access to dental care is limited (9). Tele dentistry's platforms enable early screening, risk stratification and remote triage while shifting care toward preventive and minimally invasive interventions, and reducing travel barriers and clinic congestion. When linked to epidemiological data and underpinned by public private partnerships, these digital services allow precise, scalable resource allocation that promotes equity and significantly lowers the overall oral disease burden.

Additionally, public-private partnerships can bolster these efforts, fostering collaborations that leverage diverse expertise and financial resources to implement scalable, sustainable solutions (43).

## Policy recommendations

Drawing on the pillars of sustainability, equality, and the reduction of the oral health disease burden, comprehensive policy recommendations can be advanced to transform the delivery of oral health services within public health systems (44). A foundational element of these recommendations is the development of integrated health models that seamlessly incorporate oral health into the broader framework of public healthcare (39). By doing so, oral health becomes an intrinsic component of overall health management, allowing for more coordinated care that recognizes the interconnectedness of dental conditions with chronic diseases such as diabetes and cardiovascular disorders (9, 45). Such integration not only streamlines service delivery but also facilitates the early detection and management of oral diseases within routine health check-ups, thus mitigating the progression to more severe conditions and reducing long-term healthcare costs (39, 46).

**Figure 2:**
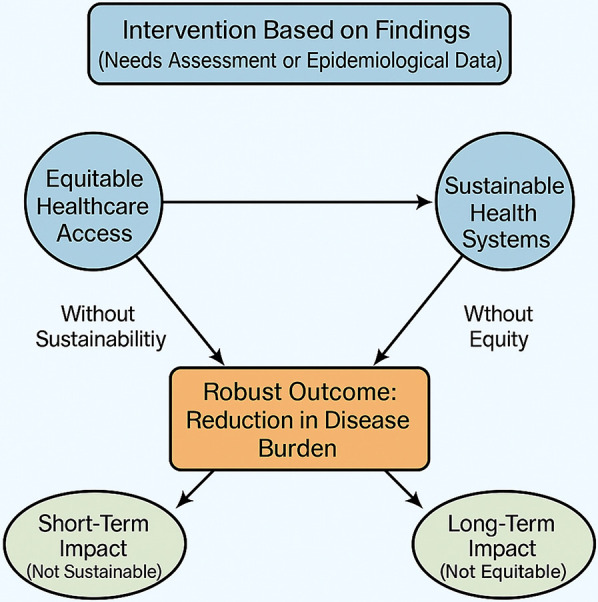
Framework for targeted oral health interventions linking needs assessment to sustainable and equitable outcomes.

Collaboration among governmental agencies, educational institutions, community organizations, and the private sector is vital to mobilizing the necessary resources and expertise (47, 48). Cross-sector partnerships enable the pooling of knowledge, technology, and financial resources, which can be leveraged to establish comprehensive care networks, especially in underserved regions (6, 20). These collaborations can also foster the development of multidisciplinary training programs that prepare healthcare professionals to address the unique challenges of oral health in diverse communities (18). These interlinked strategies are synthesized in Figure 2, which illustrates a Framework for Targeted Oral Health Interventions linking Needs Assessment to Sustainable and Equitable Outcomes. Through shared responsibility and joint initiatives, the collective expertise of these stakeholders can drive innovation in service delivery, ensuring that policies are both responsive and adaptable to evolving public health needs (4, 20).

Investment in research and innovation is crucial for developing new preventive strategies, diagnostic tools, and sustainable treatment modalities, with robust initiatives exploring emerging technologies such as digital diagnostics and tele-dentistry, which have the potential to revolutionize oral health care delivery, particularly in resource-limited settings (49). This commitment to innovation not only enhances the precision of early diagnosis but also supports the creation of interventions that are tailored to the socioeconomic contexts of various populations (4). Designing community-centered programs that are attuned to local needs further underscores the importance of equality in public health policy (50). Involving community members in the planning and execution of oral health initiatives fosters a sense of ownership and ensures that the programs address specific cultural, geographic, and socioeconomic barriers to care (20). These initiatives might include local education campaigns, the deployment of mobile dental units, and the establishment of tele-dentistry services that reach remote areas (51).

Finally, the establishment of robust monitoring and evaluation systems is essential for ensuring that policy interventions are having their intended impact and for identifying areas where continuous improvement is necessary (52, 53). Comprehensive data collection and analysis allow for real-time feedback on program effectiveness, facilitating adjustments that can optimize resource allocation and improve outcomes over time (15). Such systems not only provide accountability but also enable the dissemination of best practices across different regions and contexts (4). Regular evaluation of policy performance ensures that initiatives remain aligned with the overarching goals of sustainability, equality, and the reduction of the oral health disease burden, ultimately fostering a dynamic and responsive public health system (44, 54).

Framework for Targeted Oral Health Interventions linking Needs Assessment to Sustainable and Equitable Outcomes.

## Conclusion

Integrating the pillars of sustainability, equality, and targeted interventions against the oral health disease burden provides a robust framework for strengthening public health policies. By focusing on long-term, equitable, and high-impact strategies, policymakers can substantially enhance oral health outcomes, reduce disparities, and create resilient systems capable of addressing the future challenges of oral healthcare. This approach not only benefits individual communities but also contributes to the overall strengthening of global health systems, fostering a future where equitable oral health is within reach for all.

## Data Availability

The original contributions presented in the study are included in the article/Supplementary Material, further inquiries can be directed to the corresponding author.
